# Impact of Chronic Kidney Disease Epidemiology Collaboration (CKD-EPI) GFR Estimating Equations on CKD Prevalence and Classification Among Asians

**DOI:** 10.3389/fmed.2022.957437

**Published:** 2022-07-14

**Authors:** Bjorn Kaijun Betzler, Rehena Sultana, Feng He, Yih Chung Tham, Cynthia Ciwei Lim, Ya Xing Wang, Vinay Nangia, E. Shyong Tai, Tyler Hyungtaek Rim, Mukharram M. Bikbov, Jost B. Jonas, Se Woong Kang, Kyu Hyung Park, Ching-Yu Cheng, Charumathi Sabanayagam

**Affiliations:** ^1^Yong Loo Lin School of Medicine, National University of Singapore, Singapore, Singapore; ^2^Singapore Eye Research Institute, Singapore National Eye Centre, Singapore, Singapore; ^3^Centre for Quantitative Medicine, Duke-NUS Medical School, Singapore, Singapore; ^4^Ophthalmology and Visual Science Academic Clinical Program, Duke-NUS Medical School, Singapore, Singapore; ^5^Department of Renal Medicine, Singapore General Hospital, Singapore, Singapore; ^6^Beijing Institute of Ophthalmology, Beijing Ophthalmology & Visual Sciences Key Laboratory, Beijing Tongren Eye Center, Beijing Tongren Hospital, Capital Medical University, Beijing, China; ^7^Suraj Eye Institute, Nagpur, India; ^8^Department of Medicine, Yong Loo Lin School of Medicine, National University of Singapore, Singapore, Singapore; ^9^Department of Ophthalmology, Yonsei University College of Medicine, Seoul, South Korea; ^10^Ufa Eye Research Institute, Ufa, Russia; ^11^Department of Ophthalmology, Medical Faculty Mannheim, Heidelberg University, Mannheim, Germany; ^12^Institute of Molecular and Clinical Ophthalmology, Basel, Switzerland; ^13^Department of Ophthalmology, Samsung Medical Center, Sungkyunkwan University School of Medicine, Seoul, South Korea; ^14^Department of Ophthalmology, Seoul National University Bundang Hospital, Seoul National University College of Medicine, Seoul, South Korea

**Keywords:** chronic kidney disease, Asian, glomerular filtration rate, prevalence, epidemiology, creatinine, cystatin C

## Abstract

**Background:**

In 2021, the Chronic Kidney Disease Epidemiology Collaboration (CKD-EPI) validated a new equation for estimated glomerular filtration rate (eGFR). However, this new equation is not ethnic-specific, and prevalence of CKD in Asians is known to differ from other ethnicities. This study evaluates the impact of the 2009 and 2021 creatinine-based eGFR equations on the prevalence of CKD in multiple Asian cohorts.

**Methods:**

Eight population-based studies from China, India, Russia (Asian), Singapore and South Korea provided individual-level data (*n* = 67,233). GFR was estimated using both the 2009 CKD-EPI equation developed using creatinine, age, sex, and race (eGFRcr [2009, ASR]) and the 2021 CKD-EPI equation developed without race (eGFRcr [2021, AS]). CKD was defined as an estimated glomerular filtration rate (eGFR) <60 mL/min/1.73m2 (G3-G5). Prevalence of eGFR categories was compared within each study and within subgroups of age, sex, body mass index (BMI), diabetes, and hypertension status. The extent of reclassification was examined using net reclassification improvement (NRI).

**Findings:**

Of 67,233 adults, CKD prevalence was 8.6% (*n* = 5800/67,233) using eGFRcr (2009, ASR) and 6.4% (*n* = 4307/67,233) using eGFRcr (2021, AS). With the latter, CKD prevalence was reduced across all eight studies, ranging from −7.0% (95% CI −8.5% to −5.4%) to −0.4% (−1.3% to 0.5%), and across all subgroups except those in the BMI < 18.5% subgroup. Net reclassification index (NRI) was significant at −2.33% (*p* < 0.001). No individuals were reclassified as a higher (more severe) eGFR category, while 1.7%−4.2% of individuals with CKD were reclassified as one eGFR category lower when eGFRcr (2021, AS) rather than eGFRcr (2009, ASR) was used.

**Interpretation:**

eGFRcr (2021, AS) consistently provided reduced CKD prevalence and higher estimation of GFR among Asian cohorts than eGFRcr (2009, ASR). Based on current risk-stratified approaches to CKD management, more patients reclassified to lower-risk GFR categories could help reduce inappropriate care and its associated adverse effects among Asian renal patients. Comparison of both equations to predict progression to renal failure or adverse outcomes using prospective studies are warranted.

**Funding:**

National Medical Research Council, Singapore.

## Introduction

The glomerular filtration rate (GFR) is considered the best overall index of kidney function in medical practice (1). GFR cannot be measured easily; instead, it is common for clinical laboratories to estimate GFR using serum creatinine (eGFRcr), an endogenous filtration marker (2). Current guidelines recommend the 2009 Chronic Kidney Disease Epidemiology Collaboration (CKD-EPI) creatinine equation (1) for initial assessment of renal function (3, 4). This equation incorporates coefficients for serum creatinine, age, sex, and race (eGFRcr-ASR) (1). In 2021, a new CKD-EPI creatinine equation was validated by Inker *et al*. (5), derived from fitting the same regression function as the current equations, but without race as an explanatory variable (eGFRcr-AS) (5). Inker and colleagues (5) reported that eGFRcr-AS gave a lower estimated prevalence of chronic kidney disease (CKD) in non-black U.S. adults than eGFRcr-ASR. Because there was an insufficient representation of racial and ethnic groups other than Black and non-Black, they suggested testing this new equation in other ethnic groups (5).

The prevalence of CKD in Asians is known to differ from other ethnicities (6–9). Xu *et al*. (6) reported that native Chinese ethnicities had a significantly lower prevalence of CKD than native American ethnicities. Kramer *et al*. (7) reported that CKD prevalence varied across Caucasian, Asian, and Hispanic ethnic groups, although the degree of variability depends on the equation used to estimate GFR. In the North Indian Punjab population, Bragg-Gresham *et al*. (8) found a markedly higher prevalence of albuminuria but lower prevalence of reduced eGFR when compared against the U.S. population. Furthermore, the epidemiologic pattern and relative contribution of known CKD risk factors, such as blood pressure (10), body mass index (11), and diabetes (12), differ between Asian and Western populations.

Given that GFR estimating equations play an important role in clinical decision making, understanding how eGFRcr-AS, an equation derived from a Western population, performs in Asians is crucial. Over or underestimation of GFR may lead to misclassification of CKD status which may have implications for CKD management. Equations that underestimate GFR may overdiagnose CKD or reclassify patients to a more severe category of CKD leading to intensive management, early referrals for kidney transplant specialty care and dialysis access planning (13, 14). On the other hand, equations that overestimate GFR may underdiagnose CKD or reclassify patients to a less severe category of CKD leading to late referrals and delayed care (13, 14).

In this study, we reported comparative estimates of the prevalence of CKD and its eGFR categories, classified using both the current and new CKD-EPI creatinine equations, across multiple Asian population-based studies. We also examined whether changes in CKD prevalence varied across study cohorts, if these changes were affected by demographics, and the pattern of reclassification of eGFR categories by the 2021 equation.

## Methods

### Data Collection

The Asian Eye Epidemiology Consortium (AEEC) (15, 16) is a collaborative network of population-based studies that was established in 2018 to provide deeper insights on the trends and associated risk factors of major age-related eye diseases among Asians. The AEEC was requested to provide data on serum creatinine levels, and known risk factors of CKD such as age, sex, body mass index (BMI), presence of hypertension and presence of diabetes for each study participant. The eight participating cohorts that provided individual-level data to the data coordinating center at the Singapore Eye Research Institute were: the Beijing Eye Study (BES) from China, (17) the fifth Korea National Health and Nutrition Examination Survey (KNHANES) (18) from South Korea, the Singapore Malay Eye Study (SiMES), (19) the Singapore Indian Eye Study (SINDI) (20), the Singapore Chinese Eye Study (SCES) (20) and the Singapore Prospective Study Program (SP2) (21) from Singapore, the Ural Eye and Medical study (UEMS) (22) from Central Asia, and the Central India Eye and Medical Study (CIEMS) from India. While some of these datasets were collected by eye research institutes, they are population-based studies recruiting randomly selected participants from the community to assess the prevalence of ocular as well as non-ocular conditions. Thus, they are representative of their respective general populations. The AEEC was approved by the SingHealth Institutional Review Board in Singapore. All studies adhered to the tenets of the Declaration of Helsinki and had local ethical committee approval. All participants gave written informed consent.

### Definitions

eGFR categories were defined as (3, 23): G1 = eGFR ≥90 mL/min/1.73 m^2^; G2 = eGFR 60-89 mL/min/1.73 m^2^; G3 = eGFR 30–59 mL/min/1.73 m^2^; G4 = eGFR 15–29 mL/min/1.73 m^2^; G5 = eGFR <15 mL/min/1.73 m^2^. GFR was estimated using both the 2009 CKD-EPI Creatinine Equation (eGFRcr-ASR]) (1) and the 2021 CKD-EPI Creatinine Equation (eGFRcr-AS) (5). Presence of diabetes was identified if a random plasma glucose was ≥ 11.1 mmol/l, HbA1c was ≥ 6.5%, or participants self-reported use of hypoglycemic medication or had physician-diagnosed diabetes. Presence of hypertension was defined as systolic blood pressure of ≥ 140 mmHg, diastolic blood pressure of ≥ 90 mmHg, a self-reported history of physician-diagnosed hypertension or use of antihypertensive medication. Height was measured in centimeters using a wall-mounted measuring tape and weight in kilograms using a digital scale. BMI was calculated as the weight in kilograms divided by the square of height in meters (kg/m^2^). eGFRcr-AS and eGFRcr-ASR are governed by the following formula, albeit with different coefficients (24):


$$\begin{array}{l} eGFRcr=u\;*\;\min{\left(\frac{Scr}{k},\;1\right)}^{\alpha 1}\;*\;\max{\left(\frac{Scr}{k},\;1\right)}^{\alpha 2}\\ \;*c^{Age}\;*\;d\left[if\;female\right]\; \end{array}$$

### Statistical Analysis

The change in G3-G5 prevalence when using eGFRcr-AS compared to eGFRcr-ASR was calculated in each of the eight participating studies. This was done within the overall study cohort and within subgroups of age, sex, BMI, diabetes, and hypertension status. Change in prevalence of individual eGFR categories was also compared in each study. Net reclassification improvement (NRI) (25) was calculated to quantify the extent of reclassification when using eGFRcr-AS. Baseline characteristics of three subgroups were examined in detail: (1) participants classified as G1-G2 (eGFR ≥ 60 mL/min/1.73 m^2^) according to both equations (“Normal eGFR”); (2) participants classified as G3-G5 according to eGFRcr-ASR but reclassified as G1-G2 according to eGFRcr-AS (“Reclassified”); and (3) participants classified as G3-G5 (eGFR < 60 mL/min/1.73 m^2^) by both equations (“Non-Reclassified”). Data on serum cystatin C was only available in the SINDI and SCES cohort. Hence, in supplementary analysis, we further compared prevalence of various CKD categories in the subgroup of SINDI and SCES, using the cystatin C only equation and creatinine + cystatin C equations (eGFRcys-AS; eGFRcr-cys-ASR; eGFRcr-cys-AS). Finally, because the CKD-EPI equations were developed for black vs. non-black American populations, we further calculated G3-G5 prevalence using the modified four-level ethnicity equation proposed by Stevens et al. (26), which was a modification of eGFRcr-ASR, that significantly improved bias in Asians within their validation set. In all analyses, statistical significance was defined as *p* < 0.05. All analyses were conducted using SAS 9.4 (2016) by SAS Institute Inc., Cary, NC, USA.

## Results

**Figure 1:**
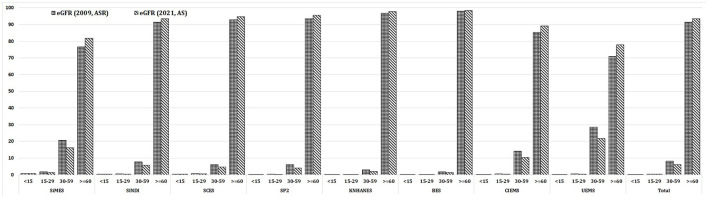
Prevalence of eGFR Categories in the eight Asian study cohorts.

Table 1 provides the baseline characteristics of participants included in the analysis of the general population which included 67,233 participants across 8 population-based studies. Prevalence of CKD (G3-G5) in the entire cohort was 8.6% (*n* = 5800/67,233) using eGFRcr-ASR and 6.4% (*n* = 4307/67,233) using eGFRcr-AS. Mean (SD) age across studies ranged from 45.7 (19.4) years (KNHANES) to 64.5 (9.6) years (BES); Proportion of men ranged from 40.9% in BES to 50.8% in SINDI. Mean (SD) BMI ranged from 19.7 (3.4) % in CIEMS to 27.9 (5.0) % in UEMS. Mean (SD) eGFR ranged from 72.0 (19.0) in UEMS to 99.1 (22.2) in KNHANES using eGFRcr-ASR, and 75.7 (19.2) in UEMS to 102.2 (20.7) in KNHANES using eGFRcr-AS. Figure 1 provides visual representation of the prevalence of each eGFR category in the eight cohorts. Table 2 shows that all 8 studies saw a reduction in CKD prevalence when eGFRcr-AS instead of eGFRcr-ASR was applied. Prevalence of CKD ranged from 2.0% (BES) to 29.1% (UEMS) using eGFRcr-ASR and 1.6% (BES) to 22.2% (UEMS) using eGFRcr-AS. The largest and smallest reduction was −7.0% (95%CI −8.5% to −5.4%) in UEMS and −0.4% (−1.3% to 0.5%) in BES respectively (Table 2). Supplementary Table 1 shows how the prevalence of each eGFR category within the individual cohorts changed from eGFRcr-ASR to eGFRcr-AS. Supplementary Table 2 further examined the changes in prevalence of eGFR categories within subgroups of age, sex, BMI, diabetes, and hypertension status. A reduction in G3-G5 prevalence when using eGFRcr-AS was observed in all subgroups, except for SCES and BES which saw no change (0.0%) in the BMI < 18.5% subgroup. The reduction in G3-G5 prevalence was also more marked in men (than women) across all included studies, except CIEMS. Supplementary Table 3 explains this observation by mathematically comparing the coefficients of the eGFRcr-ASR and eGFRcr-AS equations (24), and their net effects on the calculated eGFRcr. Table 3 reflects reclassification of eGFR categories when eGFRcr-AS was used. NRI was significant at −2.33% (*p* < 0.001). No individuals were reclassified as a higher (more severe) eGFR category, while 1,568 individuals were reclassified as a lower category. Of the 1,568 individuals 1,493 were reclassified from G3 to G1 or G2, 68 were reclassified from G4 to G3, and 7 were reclassified from G5 to G4. No participant was reclassified from G4 or G5 to G1 or G2. Table 4 examines baseline characteristics of “Normal eGFR”, “Reclassified”, and “Non-Reclassified” subgroups. There was a significant difference (all *p* < 0.001) in mean age (higher), sex (more males), mean BMI (higher), proportion with diabetes (higher) and proportion with hypertension (higher) in “Reclassified” compared to “Normal eGFR” (Table 4). Supplementary Table 4 compares prevalence of CKD categories in a subgroup cohort from SINDI and SCES, which had data on serum cystatin C. In our supplementary analysis, eGFRcys-AS estimated a 11.0% (10.2 to 11.8) prevalence of G3-G5, compared to 6.8% (6.2 to 7.5) by eGFRcr-ASR and 4.9% (4.4 to 5.5) by eGFRcr-AS. Compared to eGFRcr-ASR, eGFRcr-AS had a negative effect on G3-G5 prevalence [−1.9% (−2.2% to −1.5%)] while eGFRcys-AS has a positive effect [4.2% (3.5% to 4.9%)]. Supplementary Table 5 shows that when using the four-level ethnic variable CKD-EPI equation (26) instead of eGFRcr-ASR, all 8 studies also saw a reduction in G3-G5 prevalence. This reduction was comparable to the magnitude of reduction when eGFRcr-AS was used (Table 2).

**Table 1:** Characteristics of participants in the eight Asian study cohorts (*n* = 67233).

	**SiMES** **(*n =* 3148)**	**SINDI** **(*n =* 3259)**	**SCES** **(*n =* 3192)**	**SP2** **(*n =* 5104)**	**KNHANES** **(*n =* 35788)**	**BES** **(*n =* 1605)**	**CIEMS** **(*n =* 9296)**	**UEMS** **(n = 5841)**
Age, mean (SD)	59.2 (11.0)	57.6 (10.0)	59.5 (9.8)	50.1 (11.8)	45 (19.4)	64.5 (9.6)	49.4 (13.4)	58.9 (10.7)
Age group, years, *n* (%)								
<40	-	-	-	956 (18.7)	14510 (40.5)	-	2210 (23.8)	-
40–60	1698 (53.9)	1970 (60.5)	1761 (55.2)	3168 (62.1)	11691 (32.7)	600 (37.4)	4302 (46.3)	3187 (54.56)
>60	1450 (46.1)	1289 (39.6)	1431 (44.8)	980 (19.2)	9587 (26.8)	1005 (62.6)	2784 (30.0)	2654 (45.44)
Sex, *n* (%)								
Female	1631 (51.8)	1602 (49.2)	1600 (50.1)	2653 (52.0)	19931 (55.7)	948 (59.1)	4976 (53.5)	3291 (56.34)
Male	1517 (48.2)	1657 (50.8)	1592 (49.9)	2451 (48.0)	15857 (44.3)	657 (40.9)	4320 (46.5)	2550 (43.66)
Ethnicity	Malay	Indian	Chinese	Multi-ethnic	Korean	Chinese	Indian	Russian (Asian)
BMI, kg/m^2^	26.4 (5.1)	26.2 (4.8)	23.7 (3.6)	24.0 (4.4)	23.2 (3.6)	25.7 (3.8)	19.7 (3.4)	27.9 (5.0)
BMI Categories, *n* (%)								
<18.5 kg/m^2^	133 (4.3)	89 (2.7)	183 (5.8)	371 (7.3)	2893 (8.1)	39 (2.4)	3850 (41.4)	49 (0.84)
18.5–24.9 kg/m^2^	1182 (37.8)	1321 (40.7)	1971 (62.1)	2943 (57.8)	22410 (62.9)	696 (43.4)	4724 (50.8)	1724 (29.52)
25.0–29.9 kg/m^2^	1148 (36.7)	1280 (39.4)	849 (26.8)	1326 (26.0)	9098 (25.5)	664 (41.4)	616 (6.6)	2295 (39.29)
≥30 kg/m^2^	662 (21.2)	558 (17.2)	170 (5.4)	456 (9.0)	1241 (3.5)	206 (12.8)	104 (1.1)	1773 (30.35)
Diabetes status, *n* (%)	3148	3259	3192	5058	35383	1605	9296	5841
No	2132 (67.73)	1982 (60.82)	2627 (82.3)	4559 (90.13)	32982 (93.21)	1329 (82.8)	9058 (97.44)	5161 (88.36)
Yes	1016 (32.27)	1277 (39.18)	565 (17.7)	499 (9.87)	2401 (6.79)	276 (17.2)	238 (2.56)	680 (11.64)
Hypertension, *n* (%)	3132	3254	3189	5040	21589	1584	9296	5840
No	922 (29.44)	1300 (39.95)	1265 (39.67)	2945 (58.43)	16303 (75.52)	833 (52.59)	7213 (77.59)	3491 (59.78)
Yes	2210 (70.56)	1954 (60.05)	1924 (60.33)	2095 (41.57)	5286 (24.48)	751 (47.41)	2083 (22.41)	2349 (40.22)
eGFR, mL/min/1.73 m^2^, mean (SD)								
eGFRcr-ASR	73.8 (20.4)	86.7 (18.2)	88.5 (18.5)	87.5 (17.7)	99.1 (22.2)	95.2 (13.7)	81.0 (19.5)	72.0 (19.0)
eGFRcr-AS	77.6 (20.6)	90.7 (18.0)	92.4 (17.9)	91.1 (17.4)	102.2 (20.7)	98.7 (12.6)	84.6 (19.7)	75.7 (19.2)

*BES, Beijing Eye Study; CIEMS, Central India Eye and Medical Study; KNAHNES, Korea National Health and Nutrition Examination Survey; SCES, Singapore Chinese Eye Study; SiMES, Singapore Malay Eye Study; SINDI, Singapore Indian Eye Study; SP2, Singapore Prospective Study Program; UEMS, Ural Eye and Medical Study*.

**Table 2:** Estimated Prevalence of eGFR Categories G3-G5 (eGFR <60 mL/min/1.73 m^2^) in the study cohorts.

**Cohort**	eGFRcr–ASR*	**eGFRcr–AS**	**Change in prevalence**
	**% (95% CI)**	**% (95% CI)**	**% (95% CI)**
SiMES	23.3 (21.8 to 24.8)	18.2 (16.8 to 19.6)	−5.1 (7.1 to 3.1)
SINDI	8.5 (7.6 to 9.5)	6.4 (5.6 to 7.3)	−2.1 (−3.4 to −0.8)
SCES	7.2 (6.3 to 8.1)	5.4 (4.6 to 6.2)	−1.8 (−3.0 to −0.7)
SP2	6.4 (5.7 to 7.1)	4.5 (3.9 to 5.0)	−2.0 (−2.8 to −1.1)
KNHANES	3.1 (3.0 to 3.3)	2.2 (2.1 to 2.4)	−0.9 (−1.1 to −0.7)
BES	2.0 (1.3 to 2.7)	1.6 (1.0 to 2.3)	−0.4 (−1.3 to 0.5)
CIEMS	14.8 (14.1 to 15.5)	10.8 (10.2 to 11.5)	−4.0 (−4.9 to −3.0)
UEMS	29.1 (27.9 to 30.3)	22.2 (21.1, 23.2)	−7.0 (−8.5 to −5.4)

*BES, Beijing Eye Study; CI, Confidence Interval; CIEMS, Central India Eye and Medical Study; eGFR, estimated Glomerular Filtration Rate; KNAHNES, Korea National Health and Nutrition Examination Survey; SCES, Singapore Chinese Eye Study; SiMES, Singapore Malay Eye Study; SINDI, Singapore Indian Eye Study; SP2, Singapore Prospective Study Program; UEMS, Ural Eye and Medical Study*.

^*^Current CKD–EPI equation for Asians was developed with race as an explanatory variable, but does not include the race coefficient (as all Asians were considered “non–black”).

**Table 3:** Reclassification of eGFR categories using eGFRcr-ASR and eGFRcr-AS equations.

**eGFRcr-ASR**	**eGFRcr-AS**, ***n*** **(%)**				**Reclassified as higher category (*n*)**	**Reclassified as lower category (*n*)**	**NRI (%)**	***p***
	**G1 or G2**	**G3**	**G4**	**G5**				
G1 or G2 (*n =* 61433)	61433 (100)	-	-	-	0	1568	−2.33% (0/67233–1568/67233)	<0.0001
G3 (*n =* 5487)	1493 (27.2)	3994 (72.8)	-	-				
G4 (*n =* 226)	-	68 (30.1)	158 (69.9)	-				
G5 (*n =* 87)	-	-	7 (8)	80 (92.0)				

*eGFR, estimated Glomerular Filtration Rate; NRI, Net Reclassification Index*.

*G1 or G2 = eGFR ≥60 mL/min/ 1.73 m^2^*.

*G3 = eGFR 30-59 mL/min/ 1.73 m^2^*.

*G4 = eGFR 15-29 mL/min/ 1.73 m^2^*.

*G5 = eGFR <15 mL/min/ 1.73 m^2^*.

**Table 4:** Participant characteristics according to eGFR category reclassification.

**Characteristic**	**Normal eGFR**	**Reclassified**	**Non-Reclassified**	***p* ^a^**	***p* ^b^**
	**(*n* = 61433)**	**(*n* = 1493)**	**(*n* = 4307)**		
Age, mean (SD)	48.12 (17.00)	64.75 (11.66)	66.01 (11.51)	<0.0001	0.0330
Age group, *n* (%)				<0.0001	0.0003
<40	17569 (28.6)	45 (3.01)	62 (1.44)		
40-60	26924 (43.83)	382 (25.59)	1071 (24.87)		
>60	16940 (27.57)	1066 (71.4)	3174 (73.69)		
Sex, *n* (%)				0.0003	<0.0001
Male	28112 (45.76)	753 (50.44)	1736 (40.31)		
Female	33321 (54.24)	740 (49.56)	2571 (59.69)		
BMI, kg/m^2^, mean (SD)	23.48 (4.34)	24.55 (5.05)	24.80 (5.56)	<0.0001	0.1868
BMI Categories, *n* (%)				<0.0001	0.0100
<18.5	6865 (11.21)	173 (11.63)	569 (13.31)		
18.5–24.9	34575 (56.44)	670 (45.06)	1726 (40.36)		
25.0–29.9	15548 (25.38)	434 (29.19)	1294 (30.26)		
≥30	4273 (6.98)	210 (14.12)	687 (16.07)		
Diabetes Status, *n* (%)				<0.0001	<0.0001
No	55363 (90.74)	1231 (82.78)	3236 (75.63)		
Yes	5653 (9.26)	256 (17.22)	1043 (24.37)		
Hypertension, *n* (%)				<0.0001	<0.0001
No	32210 (67.7)	593 (43.35)	1469 (36.94)		
Yes	15369 (32.3)	775 (56.65)	2508 (63.06)		

*BMI, Body Mass Index; SD, standard deviation*.

*Normal eGFR–defined as a classification of G1-G2 according to both eGFRcr-ASR and eGFRcr-AS*.

*Reclassified—defined as a classification of G3-G5 according to eGFRcr-ASR and G1-G2 according to eGFRcr-AS*.

*Reduced eGFR—defined as a classification of G3-G5 according to both eGFRcr-ASR and eGFRcr-AS*.

*^a^For comparison of “Reclassified” vs. “Normal eGFR”*.

*^b^For comparison of “Reclassified” vs. “Reduced eGFR”*.

## Discussion

In this cross-sectional analysis including participant-level data from eight Asian cohorts, we found that CKD prevalence (G3-G5) was reduced when eGFRcr-AS was used instead of eGFRcr-ASR. This reduction occurred in all eight cohorts, and in nearly all subgroups. The largest prevalence reduction was in UEMS (-7.0%) and the smallest prevalence reduction was in BES (-0.4%). In reclassification analysis, NRI was significant at −2.33% (*p* < 0.001), no individuals were reclassified into a more severe eGFR category, and no individual in G4 or G5 based on eGFRcr-ASR was reclassified to G1 or G2.

The reduction of CKD prevalence was expected by virtue of the eGFRcr-AS and eGFRcr-ASR equations themselves. Coefficient κ remained unchanged, while the net effect of updated coefficients μ, α1, α2, and *c* act to increase eGFRcr, regardless of serum creatinine. The updated coefficient *d*, applied only to females, decreases calculated eGFRcr, but the effect of increasing μ negates the effect of reducing *d*. Hence, assuming a constant patient where age and serum creatinine remain unchanged, all calculations with eGFRcr-AS would necessarily produce a greater estimation of the GFR than eGFRcr-ASR.

The largest and smallest reduction in CKD prevalence was −7.0% in UEMS and−0.4% in BES respectively. The UEMS cohort had the highest prevalence of G3a (23.4%, *n* = 1364/5841) while BES had the lowest G3a prevalence (1.2%, *n* = 19/1605) the highest prevalence of G3a in UEMS leads to a higher proportion of individuals more likely to be reclassified from G3a to G2 when eGFRcr-AS is applied. Next, in reclassification analysis, NRI was significant at−2.33% (p<0.001). We can infer that 2.33% of individuals were classified into a lower eGFR category by eGFRcr-AS. However, NRI does not speak toward the appropriateness of the reclassification. eGFRcr-ASR could be the more suitable equation to use among Asian populations as it provides closer estimation of measured GFR in current literature. For instance, Ferreira *et al*. (27) tested both sets of equations in patients with high cardiovascular and renal risk from the Action to Control Cardiovascular Risk in Diabetes (ACCORD), Systolic Blood Pressure Intervention Trial (SPRINT), and Aldosterone Antagonist Therapy for Adults with Heart Failure and Preserved Systolic Function (TOPCAT) trials. They reported that eGFRcr-AS led to an underestimation of GFR and classified more patients as having worse CKD stages (27). Furthermore, when developing these equations, Inker *et al*. (24) reported that among non-Black participants, eGFRcr-ASR overestimated measured GFR by 0.5 (−0.9 to 0.0) mL/min/1.73 m^2^, while eGFRcr-AS overestimated measured GFR by 3.9 (−4.4 to −3.4) mL/min/1.73 m^2^. Hence, among Asians (all considered non-Black in this study), eGFRcr-ASR could perhaps provide an eGFR that more closely approximates measured GFR. If, however, the eGFRcr-AS is used instead, the present analysis showed that no participant was reclassified from G4 or G5 to G1 or G2, suggesting that a gross misclassification of renal function is unlikely.

We further examined characteristics of individuals identified as having G3-G5 under eGFRcr-ASR but G1-G2 CKD under eGFRcr-AS (“Reclassified”). All “Reclassified” individuals occurred from participants originally in G3 (*n* = 1493). “Reclassified” individuals generally had characteristics of a higher cardiovascular risk profile (28)—they were more likely to be older, male, and have higher mean BMI, diabetes, or hypertension than the “Normal eGFR” group (G1-G2 using either equation). Furthermore, those identified as G3-G5 according to both equations (“Non-Reclassified”) also had characteristics of higher cardiovascular risk than “Reclassified” individuals (more likely to be older, higher mean BMI, have diabetes, or hypertension). In short, there seems to be an increase in the cardiovascular risk profile from “Normal eGFR” to “Reclassified” to “Non-Reclassified”, in line with existing knowledge that impaired kidney function increases the risk of cardiovascular disease (28, 29). Hence, even if “Reclassified” individuals are deemed to have a better renal profile, this group of patients might still have a high cardiovascular risk requiring medical attention at an individual level. Another observation we made in our reclassification analysis was that approximately a third of G3 and G4 individuals were reclassified to a lower eGFR category by eGFRcr-AS, but individuals originally in G5 were relatively unaffected (not reclassified). Perhaps this would have more implications on reducing the healthcare burden of screening and surveillance (less frequent primary care follow up for G3 and G4 patients for progression or complications), and less impact on dialysis or transplant planning (approximately similar prevalence of G5 patients with severely impaired kidney function).

While our main analyses focused on creatinine-only equations, cystatin C is important to discuss as an alternative filtration marker for estimating GFR (24). In subgroup analysis of the SINDI and SCES cohort, both eGFRcr-ASR and eGFRcr-AS were not concordant with eGFRcys-AS in estimating absolute CKD prevalence. Compared to eGFRcr-ASR, eGFRcr-AS had a negative effect on G3-G5 prevalence while eGFRcys-AS has a positive effect. Clinicians should be aware of such nuances if opting to use these newer equations for eGFR calculation.

A strength of this study is the availability of individual patient-level data across all cohorts, allowing accurate calculation of eGFR which could influence CKD prevalence rates, subgroup, and reclassification analysis. Furthermore, our inclusion of only population-based studies may improve the validity of our findings as recruitment of stable participants from the community is less prone to selection bias. However, this study is not without its limitations. First, data on serum cystatin C levels was not available in most cohorts, preventing us from examining cystatin C or creatinine + cystatin C based eGFR equations. Although not yet widely used in clinical practice, serum cystatin C is recommended for confirmatory testing of eGFR (3). Furthermore, data on albuminuria/ UACR was also sparse. Albuminuria is needed to appropriately prognose CKD, and is frequently monitored alongside eGFR in the clinical care of patients with CKD (3). Second, there was no available data on measured GFR using exogeneous filtration markers such as iohexol, iothalamate or chromium 51-labeled ethylenediaminetetraacetic acid (^51^Cr-EDTA) (30). Obtaining measured GFR would have allowed us to validate the eGFRcr equations against measured GFR in this Asian cohort. This would lend strong evidence to the argument of whether eGFRcr-ASR or eGFRcr-AS should be used among Asian populations. Although we proposed above that eGFRcr-ASR might provide a closer estimation of actual GFR, evidence from Inker *et al*. (24) is based on non-Black individuals within the United States, which is not an ideal representation of Asian ethnicities (6). To the best of our knowledge, no studies to date have validated the new CKD-EPI equations against measured GFR in Asian populations. Alternatively, one could prospectively compare the incidence of adverse events or kidney failure in patients where eGFR was calculated with different equations, as this would be more clinically feasible.

In summary, the present analysis shows that eGFRcr-AS consistently provides a reduced CKD prevalence among Asian cohorts than eGFRcr-ASR, building on current literature which has not described the performance of this new CKD-EPI equation in Asians. Based on current risk-stratified approaches to CKD management, if eGFRcr-AS were used, more patients reclassified to lower-risk GFR categories could help reduce inappropriate care and its associated adverse effects among Asian renal patients. However, prospective studies evaluating the associations of CKD (estimated by both equations) with kidney failure or adverse outcomes are warranted to confirm if those reclassified to a lower severity level with eGFRcr-AS have a lower incidence of adverse outcomes compared to the current equation. Confirmation of our findings in prospective studies will affirm current practice that widespread use of eGFRcr-ASR in Asian healthcare institutions is appropriate, considering that CKD is a major chronic disease with substantial public health burden worldwide.

## Data Availability Statement

As the study involves human participants, the data cannot be made freely available in the manuscript, the supplemental files, or a public repository due to ethical restrictions. Nevertheless, the data are available from the Singapore Eye Research Institutional Ethics Committee for researchers who meet the criteria for access to confidential data. Interested researchers can send data access requests to the Singapore Eye Research Institute using the following email address: seri@seri.com.sg.

## Ethics Statement

The studies involving human participants were reviewed and approved by the SingHealth Institutional Review Board in Singapore. All studies adhered to the tenets of the Declaration of Helsinki and had local Ethical Committee approval. The patients/participants provided their written informed consent to participate in this study.

## Author Contributions

CS and C-YC conceived and planned the study. BKB performed the literature review and drafted the manuscript. RS and FH performed the statistical analysis. BKB, RS, FH, C-YC, and CS contributed to the interpretation of the results and additional analyses. C-YC, CS, YCT, CCL, YXW, VN, ET, THR, MMB, JBJ, SWK, and KHP provided critical feedback to the manuscript. Final version of the paper has been seen and approved by all authors. All authors contributed to the intellectual development of this paper.

## Funding

This study was supported by the National Medical Research Council, NMRC/STaR/003/2008, NMRC/0796/2003, NMRC/1249/2010, and NMRC/OFLCG/001/2017. The funders had no role in study design, data collection and analysis, decision to publish, or preparation of the manuscript.

## Conflict of Interest

The authors declare that the research was conducted in the absence of any commercial or financial relationships that could be construed as a potential conflict of interest.

## Publisher's Note

All claims expressed in this article are solely those of the authors and do not necessarily represent those of their affiliated organizations, or those of the publisher, the editors and the reviewers. Any product that may be evaluated in this article, or claim that may be made by its manufacturer, is not guaranteed or endorsed by the publisher.
