# Supplementary material for: Impact of Chronic Kidney Disease Epidemiology Collaboration (CKD-EPI) GFR Estimating Equations on CKD Prevalence and Classification Among Asians
Source: Front Med (Lausanne). 2022 Jul 14;9:957437. doi: 10.3389/fmed.2022.957437 (PMC9329617; doi:10.3389/fmed.2022.957437)
Supplement: Supplementary file 1 [file Data_Sheet_1.docx]

**SUPPLEMENTARY TABLES**

| **Supplementary Table 1. Distribution of eGFR categories by the eGFRcr-ASR and eGFRcr-AS equations** | | | | | | | | |
| --- | --- | --- | --- | --- | --- | --- | --- | --- |
|  | **eGFRcr-ASR** | | | | **eGFRcr-AS** | | | |
|  | **G3a**  **n (%)** | **G3b**  **n (%)** | **G4**  **n (%)** | **G5**  **n (%)** | **G3a**  **n (%)** | **G3b**  **n (%)** | **G4**  **n (%)** | **G5**  **n (%)** |
| SIMES | 466 (14.8) | 187 (5.9) | 55 (1.8) | 25 (0.8) | 370 (11.8) | 138 (4.4) | 43 (1.4) | 22 (0.7) |
| SINDI | 189 (5.8) | 64 (2) | 15 (0.5) | 10 (0.3) | 140 (4.3) | 47 (1.4) | 13 (0.4) | 10 (0.3) |
| SCES | 152 (4.8) | 46 (1.4) | 21 (0.7) | 11 (0.3) | 101 (3.2) | 45 (1.4) | 15 (0.5) | 10 (0.3) |
| SP2 | 256 (5.0) | 53 (1.0) | 13 (0.3) | 5 (0.1) | 171 (3.4) | 41 (0.8) | 10 (0.2) | 5 (0.1) |
| KNHANES | 877 (2.5) | 177 (0.5) | 51 (0.1) | 19 (0.1) | 616 (1.7) | 127 (0.4) | 38 (0.1) | 17 (0.1) |
| BES | 19 (1.2) | 9 (0.6) | 1 (0.1) | 3 (0.2) | 16 (1.0) | 6 (0.4) | 1 (0.1) | 3 (0.2) |
| CIMES | 1064 (11.5) | 258 (2.8) | 44 (0.5) | 10 (0.1) | 784 (8.4) | 186 (2) | 28 (0.3) | 10 (0.1) |
| UEMS | 1364 (23.4) | 306 (5.2) | 26 (0.5) | 4 (0.1) | 1057 (18.1) | 217 (3.7) | 17 (0.3) | 3 (0.1) |
| Overall | 4387 (6.5) | 1100 (1.6) | 226 (0.3) | 87 (0.1) | 3255 (4.8) | 807 (1.2) | 165 (0.2) | 80 (0.1) |
| **Abbreviations:** BES, Beijing Eye Study; BMI, Body Mass Index; CIEMS, Central India Eye and Medical Study; eGFR, estimated Glomerular Filtration Rate; KNAHNES, Korea National Health and Nutrition Examination Survey; SCES, Singapore Chinese Eye Study; SiMES, Singapore Malay Eye Study; SINDI, Singapore Indian Eye Study; SP2, Singapore Prospective Study Program; UEMS, Ural Eye and Medical Study;  Percentages are given as a proportion of each cohort’s entire population under study (CKD and non-CKD)  G3a= eGFR 45-59 mL/min/ 1.73 m^2^  G3b= eGFR 30-44 mL/min/ 1.73 m^2^  G4= eGFR 15-29 mL/min/ 1.73 m^2^  G5= eGFR<15 mL/min/ 1.73 m^2^ | | | | | | | | |

| **Supplementary Table 2. Frequencies of eGFR categories G3-G5 (%) using eGFRcr-ASR and eGFRcr-AS equations, in demographic subgroups** | | | | | | | | |
| --- | --- | --- | --- | --- | --- | --- | --- | --- |
|  | SiMES  (n=3148) | SINDI  (n= 3259) | SCES  (n= 3192) | SP2  (n= 5104) | KNHANES  (n= 35788) | BES  (n= 1605) | CIEMS  (n= 9296) | UEMS  (n = 5841) |
| eGFR<60 (G3-G5), n (%) |  |  |  |  |  |  |  |  |
| All adults |  |  |  |  |  |  |  |  |
| eGFRcr-ASR | 733 (23.3) | 278 (8.5) | 230 (7.2) | 327 (6.4) | 1124 (3.1) | 32 (2.0) | 1376 (14.8) | 1700 (29.1) |
| eGFRcr-AS | 573 (18.2) | 210 (6.4) | 171 (5.4) | 227 (4.5) | 798 (2.2) | 26 (1.6) | 1008 (10.8) | 1294 (22.2) |
| Change in % | -5.1 (-7.1, -3.1) | -2.1 (-3.4, -0.8) | -1.8 (-3.0, -0.7) | -2.0 (-2.8, -1.1) | -0.9 (-1.1, -0.7) | -0.4 (-1.3, 0.5) | -4.0 (-4.9, -3.0) | -7.0 (-8.5, -5.4) |
| Age <60 years |  |  |  |  |  |  |  |  |
| eGFRcr-ASR | 7.7 (6.4, 9.0) | 2.4 (1.7, 3.1) | 1.3 (0.7, 1.9) | 2.6 (2.1, 3.1) | 0.4 (0.3, 0.5) | - | 7.7 (7.1, 8.4) | 20.0 (18.6, 21.4) |
| eGFRcr-AS | 5.1 (4.0, 6.1) | 1.8 (1.2, 2.4) | 0.9 (0.4, 1.4) | 1.7 (1.3, 2.1) | 0.3 (0.2, 0.4) | - | 5.7 (5.1, 6.2) | 14.8 (13.6, 16.1) |
| Change in % | -2.6 (-4.2, -1.0) | -0.6 (-1.5, 0.3) | -0.4 (-1.1, 0.3) | -0.9 (-1.6, -0.3) | -0.1 (-0.2, 0.0) | - | -2.1 (-2.9, -1.2) | -5.2 (-7.0, -3.3) |
| Age ≥60 years |  |  |  |  |  |  |  |  |
| eGFRcr-ASR | 41.6 (39.0, 44.2) | 17.9 (15.8, 20.1) | 14.5 (12.6, 16.3) | 22.2 (19.6, 24.9) | 10.6 (10.0, 11.2) | 3.2 (2.1, 4.3) | 31.3 (29.6, 33.1) | 40.1 (38.2, 42.0) |
| eGFRcr-AS | 33.6 (31.1, 36.1) | 13.5 (11.6, 15.4) | 10.8 (9.2, 12.5) | 15.9 (13.6, 18.3) | 7.4 (6.9, 8.0) | 2.6 (1.6, 3.6) | 23.0 (21.4, 24.6) | 31.0 (29.2, 32.8) |
| Change in % | -8.0 (-11.5, -4.5) | -4.4 (-7.2, -1.6) | -3.6 (-6.1, -1.2) | -6.3 (-9.8, -2.9) | -3.1 (-3.9, -2.3) | -0.6 (-2.1, 0.9) | -8.3 (-10.7, -6.0) | -9.1 (-11.7, -6.6) |
| Men |  |  |  |  |  |  |  |  |
| eGFRcr-ASR | 25.2 (23.0, 27.5) | 8.8 (7.4, 10.1) | 9.9 (8.4, 11.4) | 8.1 (7.0, 9.2) | 3.3 (3.1, 3.6) | 2.0 (0.8, 3.1) | 12.5 (11.6, 13.6) | 20.4 (18.8, 22.0) |
| eGFRcr-AS | 18.6 (16.6, 20.6) | 6.4 (5.2, 7.6) | 6.8 (5.6, 8.1) | 5.4 (4.5, 6.3) | 2.3 (2.1, 2.6) | 1.7 (0.6, 2.7) | 9.0 (8.1, 9.9) | 13.3 (12.0, 14.7) |
| Change in % | -6.7 (-9.6, -3.7) | -2.4 (-4.2, -0.6) | -3.1 (-5.0, -1.2) | -2.7 (-4.1, -1.3) | -1.0 (-1.4, -0.7) | -0.3 (-1.8, 1.1) | -3.6 (-4.9, -2.3) | -7.1 (-9.1, -5.0) |
| Women |  |  |  |  |  |  |  |  |
| eGFRcr-ASR | 21.5 (19.4, 23.5) | 8.3 (6.9, 9.7) | 4.5 (3.5, 5.6) | 4.8 (4.0, 5.7) | 3.0 (2.8, 3.2) | 2.0 (0.8, 3.1) | 16.8 (15.7, 17.8) | 35.9 (34.2, 37.5) |
| eGFRcr-AS | 17.8 (16.0, 19.7) | 6.5 (5.3, 7.7) | 3.9 (2.9, 4.9) | 3.5 (2.8, 4.3) | 2.2 (2.0, 2.4) | 1.7 (0.6, 2.7) | 12.5 (11.5, 13.4) | 29.0 (27.4, 30.6) |
| Change in % | -3.6 (-6.3, -0.9) | -1.8 (-3.6, 0.0) | -0.6 (-2.0, 0.8) | -1.3 (-2.4, -0.2) | -0.8 (-1.1, -0.5) | -0.3 (-1.8, 1.1) | -4.3 (-5.7, -2.9) | -4.3 (-5.7, -2.9) |
| BMI <18.5% |  |  |  |  |  |  |  |  |
| eGFRcr-ASR | 15.0 (8.6, 21.5) | 11.2 (4.1, 18.4) | 3.8 (0.8, 6.9) | 3.8 (1.7, 5.9) | 1.5 (1.1, 2.0) | 7.7 (0.0, 17.3) | 16.5 (15.3, 17.7) | 16.3 (5.0, 27.7) |
| eGFRcr-AS | 12.0 (6.1, 17.9) | 9.0 (2.5, 15.5) | 3.8 (0.8, 6.9) | 3.0 (1.1, 4.8) | 1.2 (0.8, 1.7) | 7.7 (0.0, 17.3) | 12.5 (11.5, 13.6) | 12.2 (2.1, 22.4) |
| Change in % | -3.0 (-11.2, 5.2) | -2.2 (-11.1, 6.6) | 0.0 (-3.9, 3.9) | -0.8 (-3.4, 1.8) | -0.3 (-0.9, 0.3) | 0.0 (-11.8, 11.8) | -4.0 (-5.6, -2.4) | -4.1 (-17.9, 9.8) |
| BMI 18.5–24.9% |  |  |  |  |  |  |  |  |
| eGFRcr-ASR | 23.3 (20.8, 25.7) | 7.6 (6.2, 9.1) | 6.1 (5.1, 7.2) | 5.8 (5.0, 6.7) | 2.7 (2.5, 2.9) | 3.3 (1.9, 4.7) | 13.5 (12.5, 14.5) | 26.7 (24.6, 28.8) |
| eGFRcr-AS | 17.8 (15.6, 20.0) | 6.4 (5.1, 7.8) | 4.6 (3.7, 5.6) | 4.0 (3.3, 4.8) | 1.8 (1.7, 2.0) | 2.6 (1.3, 3.8) | 9.5 (8.7, 10.4) | 19.7 (17.8, 21.6) |
| Change in % | -5.5 (-8.7, -2.3) | -1.2 (-3.2, 0.7) | -1.5 (-2.9, -0.1) | -1.8 (-2.9, -0.7) | -0.9 (-1.1, -0.6) | -0.7 (-2.5, 1.1) | -3.9 (-5.2, -2.7) | -7.0 (-9.8, -4.2) |
| BMI 25.0-29.9% |  |  |  |  |  |  |  |  |
| eGFRcr-ASR | 24.0 (21.5, 26.6) | 8.0 (6.5, 9.6) | 9.7 (7.6, 11.7) | 8.0 (6.5, 9.5) | 4.4 (4.0, 4.9) | 0.9 (0.1, 1.7) | 13.3 (10.6, 16.1) | 29.2 (27.3, 31.0) |
| eGFRcr-AS | 18.3 (16.0, 20.6) | 5.2 (4.0, 6.5) | 6.9 (5.2, 8.7) | 5.9 (4.6, 7.2) | 3.3 (2.9, 3.6) | 0.8 (0.0, 1.5) | 9.7 (7.3, 12.2) | 22.6 (20.9, 24.4) |
| Change in % | -5.7 (-9.1, -2.4) | -2.8 (-4.7, -0.9) | -2.7 (-5.3, -0.1) | -2.1 (-4.0, -0.2) | -1.2 (-1.7, -0.6) | -0.2 (-1.1, 0.8) | -3.6 (-7.1, 0.0) | -6.5 (-9.1, -4.0) |
| BMI ≥30% |  |  |  |  |  |  |  |  |
| eGFRcr-ASR | 22.5 (19.3, 25.8) | 10.6 (7.9, 13.2) | 8.8 (4.3, 13.4) | 7.2 (4.8, 9.7) | 4.5 (3.3, 5.7) | - | 21.2 (12.8, 29.5) | 31.8 (29.6, 34.0) |
| eGFRcr-AS | 19.0 (16.0, 22.1) | 8.1 (5.7, 10.4) | 5.9 (2.1, 9.7) | 3.7 (1.9, 5.6) | 3.5 (2.4, 4.5) | - | 15.4 (8.0, 22.8) | 24.3 (22.2, 26.3) |
| Change in % | -3.5 (-7.8, 0.9) | -2.5 (-5.9, 0.9) | -2.9 (-8.5, 2.6) | -3.5 (-6.5, -0.6) | -1.0 (-2.6, 0.5) | - | -5.8 (-16.2, 4.7) | -7.5 (-10.4, -4.6) |
| Diabetes Status |  |  |  |  |  |  |  |  |
| eGFRcr-ASR | 34.5 (31.6, 37.5) | 13.5 (11.6, 15.4) | 14 (11, 16.9) | 17.2 (13.8, 20.7) | 13.5 (12.1, 14.8) | 4.3 (1.8, 6.9) | 24.8 (19.1, 30.5) | 31.9 (28.3, 35.5) |
| eGFRcr-AS | 29.0 (26.2, 31.9) | 10.3 (8.6, 12.1) | 11.3 (8.6, 14.0) | 13.8 (10.7, 17.0) | 10.5 (9.3, 11.8) | 3.6 (1.2, 6.0) | 20.2 (14.9, 25.5) | 25.3 (22.0, 28.6) |
| Change in % | -5.5 (-9.6, -1.5) | -3.1 (-5.6, -0.6) | -2.7 (-6.5, 1.2) | -3.4 (-7.9, 1.1) | -2.9 (-4.8, -1.1) | -0.7 (-4.0, 2.5) | -4.6 (-12.1, 2.9) | -6.6 (-11.4, -1.8) |
| Non-Diabetic |  |  |  |  |  |  |  |  |
| eGFRcr-ASR | 17.9 (16.3, 19.6) | 5.3 (4.3, 6.4) | 5.7 (4.8, 6.7) | 5.3 (4.6, 5.9) | 2.3 (2.2, 2.5) | 1.5 (0.8, 2.2) | 14.5 (13.8, 15.3) | 28.7 (27.5, 30) |
| eGFRcr-AS | 13.0 (11.6, 14.5) | 3.9 (3.1, 4.8) | 4.1 (3.3, 4.9) | 3.4 (2.9, 4.0) | 1.6 (1.4, 1.7) | 1.2 (0.6, 1.8) | 10.6 (10, 11.2) | 21.7 (20.6, 22.9) |
| Change in % | -4.9 (-7.0, -2.7) | -1.4 (-2.7, -0.1) | -1.7 (-2.8, -0.5) | -1.8 (-2.7, -1.0) | -0.8 (-1.0, -0.5) | -0.3 (-1.2, 0.6) | -3.9 (-4.9, -3.0) | -7.0 (-8.7, -5.3) |
| Hypertension |  |  |  |  |  |  |  |  |
| eGFRcr-ASR | 30.4 (28.4, 32.3) | 12.7 (11.2, 14.3) | 11.1 (9.6, 12.5) | 12.5 (11.1, 14.0) | 9.3 (8.5, 10.1) | 3.3 (2.0, 4.7) | 26.0 (24.1, 27.9) | 35.4 (33.5, 37.4) |
| eGFRcr-AS | 24.4 (22.6, 26.2) | 9.8 (8.4, 11.1) | 8.4 (7.2, 9.7) | 8.9 (7.6, 10.1) | 6.5 (5.8, 7.2) | 2.7 (1.5, 3.9) | 19.7 (18.0, 21.4) | 27.9 (26.1, 29.8) |
| Change in % | -6.0 (-8.6, -3.3) | -3.0 (-4.9, -1.0) | -2.7 (-4.5, -0.8) | -3.6 (-5.5, -1.8) | -2.8 (-3.8, -1.7) | -0.7 (-2.4, 1.1) | -6.3 (-8.8, -3.7) | -7.5 (-10.1, -4.8) |
| No Hypertension |  |  |  |  |  |  |  |  |
| eGFRcr-ASR | 6.4 (4.8, 8.0) | 2.2 (1.4, 3.1) | 1.3 (0.7, 2.0) | 2.2 (1.6, 2.7) | 1.1 (1.0, 1.3) | 0.8 (0.2, 1.5) | 11.6 (10.8, 12.3) | 24.9 (23.4, 26.3) |
| eGFRcr-AS | 3.6 (2.3, 4.8) | 1.5 (0.8, 2.2) | 0.7 (0.2, 1.2) | 1.4 (0.9, 1.8) | 0.8 (0.6, 0.9) | 0.7 (0.1, 1.4) | 8.3 (7.7, 8.9) | 18.3 (17.0, 19.6) |
| Change in % | -2.8 (-4.8, -0.8) | -0.8 (-1.8, 0.3) | -0.6 (-1.4, 0.2) | -0.8 (-1.5, -0.1) | -0.3 (-0.6, -0.1) | -0.1 (-1.0, 0.7) | -3.3 (-4.3, -2.3) | -6.6 (-8.5, -4.7) |
| **Abbreviations:** BES, Beijing Eye Study; BMI, Body Mass Index; CIEMS, Central India Eye and Medical Study; GFR, Glomerular Filtration Rate; KNAHNES, Korea National Health and Nutrition Examination Survey; SCES, Singapore Chinese Eye Study; SD, standard deviation; SiMES, Singapore Malay Eye Study; SINDI, Singapore Indian Eye Study; SP2, Singapore Prospective Study Program; UEMS, Ural Eye and Medical Study; | | | | | | | | |

| **Supplementary Table 3.** **Net effect of 2009 and 2021 coefficients on eGFRcr calculation** | | | | | | | |
| --- | --- | --- | --- | --- | --- | --- | --- |
|  | **µ** | **ĸ** | **α1** | **α2** | ***c*** | ***d*** | **Net effect** |
| eGFRcr-ASR | 141 | Female: 0.7  Male: 0.9 | Female: -0.329  Male: -0.411 | -1.209 | 0.9929 | 1.018 |  |
| eGFRcr-AS | 142 | Female: 0.7  Male: 0.9 | Female: -0.241  Male: -0.302 | -1.200 | 0.9938 | 1.012 |  |
| Effect on eGFRcr | Increase | Unchanged | Increase | Increase | Increase | Decrease (if female) | Increase |

| **Supplementary Table 4.** **Estimated Prevalence of eGFR and Albuminuria categories among SINDI and SCES adults, with cystatin C equations** | | | | |
| --- | --- | --- | --- | --- |
| **Equation** | **Prevalence, % (95%CI)** | | | **Change in combined prevalence**  **(95% CI)** |
|  | **SINDI**  **(n=2919)** | **SCES**  **(n=2716)** | **Combined***  **(n=****5635)** |  |
| **G3-G5** |  |  |  |  |
| eGFRcr-ASR | 7.6 (6.7 to 8.6) | 5.9 (5 to 6.8) | 6.8 (6.2 to 7.5) | Reference |
| eGFRcr-AS | 5.4 (4.6 to 6.3) | 4.4 (3.6 to 5.1) | 4.9 (4.4 to 5.5) | -1.9 (-2.2 to -1.5) |
| eGFRcys-AS | 14.3 (13 to 15.6) | 7.4 (6.4 to 8.4) | 11.0 (10.2 to 11.8) | 4.2 (3.5 to 4.9) |
| eGFRcr-cys-ASR | 9.1 (8.1 to 10.1) | 5.5 (4.7 to 6.4) | 7.4 (6.7 to 8.1) | 0.6 (0.1 to 1) |
| eGFRcr-cys-AS | 7.6 (6.6 to 8.5) | 4.5 (3.7 to 5.3) | 6.1 (5.5 to 6.7) | -0.7 (-1.2 to -0.3) |
| **G3** |  |  |  |  |
| eGFRcr-ASR | 7.1 (6.1 to 8) | 5.2 (4.3 to 6) | 6.2 (5.5 to 6.8) | Reference |
| eGFRcr-AS | 4.9 (4.2 to 5.7) | 3.8 (3.1 to 4.5) | 4.4 (3.9 to 4.9) | -1.8 (-2.1 to -1.4) |
| eGFRcys-AS | 13.3 (12.1 to 14.6) | 6.4 (5.5 to 7.3) | 10 (9.2 to 10.8) | 3.8 (3.1 to 4.5) |
| eGFRcr-cys-ASR | 8.4 (7.4 to 9.4) | 4.7 (3.9 to 5.5) | 6.6 (6 to 7.3) | 0.4 (0 to 0.9) |
| eGFRcr-cys-AS | 7 (6 to 7.9) | 3.9 (3.1 to 4.6) | 5.5 (4.9 to 6) | -0.7 (-1.2 to -0.2) |
| **G4** |  |  |  |  |
| eGFRcr-ASR | 0.4 (0.2 to 0.7) | 0.6 (0.3 to 0.9) | 0.5 (0.3 to 0.7) | Reference |
| eGFRcr-AS | 0.4 (0.2 to 0.6) | 0.4 (0.2 to 0.6) | 0.4 (0.2 to 0.5) | -0.1 (-0.2 to 0) |
| eGFRcys-AS | 0.8 (0.5 to 1.2) | 0.9 (0.5 to 1.2) | 0.9 (0.6 to 1.1) | 0.3 (0.1 to 0.5) |
| eGFRcr-cys-ASR | 0.6 (0.3 to 0.9) | 0.8 (0.4 to 1.1) | 0.7 (0.5 to 0.9) | 0.2 (0 to 0.3) |
| eGFRcr-cys-AS | 0.5 (0.2 to 0.7) | 0.5 (0.2 to 0.8) | 0.5 (0.3 to 0.7) | 0 (-0.1 to 0.1) |
| **G5** |  |  |  |  |
| eGFRcr-ASR | 0.1 (0 to 0.3) | 0.1 (0 to 0.3) | 0.1 (0 to 0.2) | Reference |
| eGFRcr-AS | 0.1 (0 to 0.3) | 0.1 (0 to 0.3) | 0.1 (0 to 0.2) | 0 (0 to 0) |
| eGFRcys-AS | 0.1 (0 to 0.3) | 0.1 (0 to 0.3) | 0.1 (0 to 0.2) | 0 (-0.1 to 0.1) |
| eGFRcr-cys-ASR | 0.1 (0 to 0.3) | 0.1 (0 to 0.2) | 0.1 (0 to 0.2) | 0 (-0.1 to 0) |
| eGFRcr-cys-AS | 0.1 (0 to 0.3) | 0.1 (0 to 0.2) | 0.1 (0 to 0.2) | 0 (-0.1 to 0) |
| **Abbreviations:** eGFR, estimated Glomerular Filtration Rate; SCES, Singapore Chinese Eye Study; SINDI, Singapore Indian Eye Study;  G3= eGFR 30-59 mL/min/1.73 m^2^  G4= eGFR 15-29 mL/min/1.73 m^2^  G5= eGFR <15 mL/min/1.73 m^2^  *1015 participants were excluded for missing data on cystatin C, of which 302 participants additionally did not have creatinine records. | | | | |

| **Supplementary Table 5. Estimated Prevalence of eGFR Categories G3-G5 (eGFR <60 mL/min/1.73 m^2^) using the 4-level ethnic variable equation** | | | |
| --- | --- | --- | --- |
| **Cohort** | **eGFRcr-ASR**  **% (95% CI)** | **4- level ethnic variable CKD-EPI***  **% (95% CI)** | **Change in prevalence**  **% (95% CI)** |
| SiMES | 23.3 (21.8 to 24.8) | 18.7 (17.3 to 20.0) | -4.6 (-6.6 to -2.6) |
| SINDI | 8.5 (7.6 to 9.5) | 6.6 (5.7 to 7.4) | -2 (-3.4 to -0.7) |
| SCES | 7.2 (6.3 to 8.1) | 5.6 (4.8 to 6.4) | -1.6 (-2.8 to -0.4) |
| SP2 | 6.4 (5.7 to 7.1) | 4.6 (4.0 to 5.2) | -1.8 (-2.7 to -0.9) |
| KNHANES | 3.1 (3.0 to 3.3) | 2.3 (2.2 to 2.5) | -0.8 (-1.1 to -0.6) |
| BES | 2.0 (1.3 to 2.7) | 1.6 (1.0 to 2.3) | -0.4 (-1.3 to 0.5) |
| CIEMS | 14.8 (14.1 to 15.5) | 11.3 (10.7 to 12.0) | -3.5 (-4.5 to -2.5) |
| UEMS | 29.1 (27.9, 30.3) | 22.4 (21.3 to 23.5) | -6.7 (-8.3 to -5.1) |
| **Abbreviations:** BES, Beijing Eye Study; CI, Confidence Interval; CIEMS, Central India Eye and Medical Study; eGFR, estimated Glomerular Filtration Rate; KNAHNES, Korea National Health and Nutrition Examination Survey; SCES, Singapore Chinese Eye Study; SiMES, Singapore Malay Eye Study; SINDI, Singapore Indian Eye Study; SP2, Singapore Prospective Study Program; UEMS, Ural Eye and Medical Study;  * 4- level ethnic variable CKD-EPI equation: eGFR = 141 × min(Scr/k, 1)^α^ × max(Scr/k, 1)^-1.210^ × 0.993^Age^ × 0.993 [if female] × 1.16 [if Black] × 1.05 [if Asian] × 1.01 [if Hispanic and Native American] where Scr is serum creatinine, k is 0.7 for females and 0.9 for males, α is -0.328 for females and -0.412 for males, min indicates the minimum of Scr/k or 1, and max indicates the maximum of Scr/k. | | | |
